# Louisiana State Dental Society

**Published:** 1886-06

**Authors:** 


### THE DENTAL REGISTER.

Vol. XL.] JUNE, 1886. [No. 6.

### Proceedings of Societies.

### Louisiana State Dental Society.

ANNUAL MEETING, 1886.

(Reported for the Dental Register by Mrs. M. W. J.)

The annual meeting of the Louisiana State Dental Society was held in Tulane Hall, New Orleans, March 4, 5, 6, 1886.

Col. Wm. Preston Johnston, President of Tulane University, delivered

### AN ADDRESS OF WELCOME.

He said that he regretted that most pressing engagements had rendered it impossible for him to prepare an address worthy of the occasion, as it was more pleasant to listen to a speaker than to a reader. He would have preferred to deliver an extemporaneous address, but it was a well-known fact that it required much more time and labor to prepare an "extemporaneous" address than written remarks. He would, therefore, have to read what he would have preferred to have said. He gave the members of the Society a hearty welcome to Tulane University, and hoped that they would always gather there for educational purposes. That education was the great civilizing and lifting power. That in their relation to education they should consider what education was doing for them, and what they were doing for education. That education had made dentistry what it now is—one of the most honored and honorable professions—requiring the exercise of all the faculties of the mind, in combination with the highest mechanical skill and ingenuity, for the alleviation of human suffering and the promotion of health; the man must be stupid who would underrate the importance of the profession—a profession to which all the arts and sciences were tributary, even electricity being called to do its behests. He spoke of the great benefit of association in working together for good, looking to a higher standard of attainment. He assured the members of the Society that anything that Tulane University could do, would be done to further the advancement of the dental profession of Louisiana. He suggested, as a practical plan, the institution in Tulane University of a one year's course, preparatory to the dental college, to include English, biology, physiology, physics, mechanical drawing, and manual training—all to be taught, not by ordinary men, but by the ablest men that could be gathered together from the highest institutes of learning. Students who had taken such a course as this would be better prepared to enter the dental college. He hoped the profession would indorse this suggestion and urge its adoption. They might not at first see the benefit of the class in manual training, but that by it the fingers would be trained to that delicacy of touch, finer even than required for lace-work, so essential in their profession; the eye would also be trained to accurate measurement, and judgment of color and form. They would be taught the working and shaping of wood and of metals, both soft and hard; also the plastic arts. The Medical College of the University was about to adopt this course—not compelling but urging students to make it a preparatory year for the regular course, and he desired to extend its advantages to dental students, for whom it was specially desirable. To a learned divine who had objected that his sons did not require such training, wanting only abstract thought, he had replied: "You are above your vocation; you forget that your Master was a carpenter!" The best thoughts were those which were wrought into deeds; the most profound thought into action. In conclusion, he once more bade them a hearty welcome to Tulane Hall, and all its advantages, now and always.

After the transaction of the usual routine business of organization, several interesting communications were read.

One from Dr. L. C. Anderson (Lake Charles, La.) gave the history of a case of

ANTRAL CATARRH

which after unsuccessful treatment for six or seven months with iodoform, boracic acid, carbolic acid, etc., finally yielded to two weeks' daily treatment with a solution of bi-chloride of mercury, 2 grains to the oz. One grain to the oz. used for a week having failed to benefit. There had been no relapse at the expiration of a year. The case had been under treatment by the physician for three years previous to coming to the dentist.

DISCUSSION.

*Dr. J. S. Knapp* said that the treatment with corrosive sublimate was new to him; that but comparatively few cases of antral diseases were permanently cured, both patient and operator becoming discouraged by the long-continued treatment necessary under the old methods; has had some success with wood creosote, three drops to a teaspoonful of alcohol in 2 oz. water, injected daily—inquired whether a canula was generally used? Treatment with aromatic sulph. acid good when there was necrosed bone, in necrosed alveolar process two applications, four or five days apart would often prove sufficient. A diseased condition of the antrum was often induced by a dead molar of which the roots had penetrated the floor of the antrum; in that case the tooth should be removed and the antrum treated through the opening made by the roots.

*Dr. A. G. Friedrichs* endorsed the treatment with bichloride of mercury, which was a powerful germicide and disinfectant; that the essential points were drainage and absolute cleanliness; failures were due to patients failing to follow directions.

*Dr. D. G. Parker* (Opelousas, La.) treated with 20 per cent. solution of elixir vitriol. Described an interesting case in which necrosed alveolar process followed an attack of typhoid fever. All of the teeth back of the cuspid were lost, leaving an opening through which three fingers could be passed. The cavity was syringed daily, as above, and applications of the same remedy made on pellets of cotton laid in the cavity; loose pieces of bone were removed for nine months, many through the nose. A perfect cure was finally effected. Elixir vitriol was the only remedy used.

*Dr. G. J. Friedrichs* (from the chair) said that a congested or inflamed catarrhal condition, due to common cold which would cure itself, and needed no treatment, was often mistaken for antral trouble successfully treated; that it was not advisable to confine treatment to any one remedy; there were hundreds by which the same result could be attained. Subject passed.

*Dr. Oscar Salomon* read a paper entitled

PHYSICIAN AND DENTIST, a discussion of the relationship between the professions of medicine and dentistry; how far the province of the dental surgeon extends, and when the physician should be appealed to, and *vice versa*; pointing out the defects and wrongs of the present relative position of the two professions, and suggesting remedies for the same.

He said that physicians, as a rule, send patients to the dentist only when they are at a loss what to do—as when treatment for *mumps* fails to give relief, the real trouble being a *parulis*; or after treating in vain *a tumor* which proves to be a small *exostosis*.

He cited a recent case in his practice—a young girl of 13, who had been confined to her bed for nearly two weeks with mumps—as pronounced by the attending physician. For two days she had been unable to open her mouth sufficiently for the introduction of the handle of a spoon. She had been tortured by hot poultices, but the swelling continued to increase rather than diminish. He ordered cold applications in order to relax the rigidity of the muscles, but on his return in the afternoon, found the case worse than ever, the attending physician having called in the meantime and forbidden the cold applications as imperiling the life of the patient. The mother was finally induced to follow the directions of the dentist. As the result of cold applications externally, with listerine as a mouth-wash, introduced by means of a small syringe, and quinine at night, the jaws were so far relaxed by the next morning that the introduction of the little finger was possible, revealing a large swelling extending from the first lower right bicuspid to the second molar. A 25 per cent. solution of cocaine (Merck's) was applied, and with a Stellwagen upper separating forceps, the beak of which was covered with gutta-percha, the mouth was opened without much pain, and two small temporary roots found wedged between the second bicuspid and first molar. Their removal was followed by a profuse discharge of pus, and the mumps disappeared in 24 hours.

Another case had been treated for several months for a small tumor underneath the ear. The teeth were apparently faultless, with the exception of the upper right wisdom tooth, which was slightly decayed, the position of the crown being somewhat abnormal, diverging outwardly. Tracing the direction of the roots with a fine flexible steel probe, a somewhat softened mass, not unlike macerated bone, was revealed. Concluding that the soft osseous deposit, together with exostosis, formed the tumor-like swelling, the patient was persuaded to have the wisdom-tooth extracted, when the *tumor* disappeared.

Had the physician possessed more knowledge of oral surgery, or the courage to admit the lack of it, and a dentist been at once consulted, the patient would have been spared much agony of mind in anticipation of a torturing operation.

True dentistry is a specialty of medicine. Not the trade of tooth-pulling, nor the men who parade in the newspapers as—"The only man with the gas engine;" the "Hypnotic Air" quack; "The Champion Exposition Dentist;" Better than the Best—Cheaper than the Cheapest." Such men have degraded our profession in the eyes, not only of the general public, but especially of the medical profession, so that the latter are justly afraid and ashamed to acknowledge us as brethren.

A dentist, to be worthy of the name, must not only be able to make beautiful fillings, but he must have a knowledge of the causes of caries, and should understand thoroughly pathology, physiology, and materia medica. Without this he will be ignorant, for example, of the primary causes of the swelling of the gums in our girl-patients, at the time of puberty, or when menstruation is obstructed, and in his ignorance might scarify or even cauterize the gum. Without this knowledge he would not understand the lack of nourishment of pregnant or nursing patients, or know how to treat the marvellous changes in the saliva of such patients.

He requires the same degree of intelligence and judgment as the oculist or the aurist; and when this is the case, he is as intimately related to medicine as they are.

The remedy for these defects and wrongs must be based upon the present conditions. Dr. Chapin A. Harris was right when he said that the diseases and treatment of the teeth should be taught in medical colleges, and that dental surgery should be a specialty of medicine. It was only when the University of Maryland refused to establish special chairs for that purpose, he joined with others, in 1839, in the establishment of the first dental college in the world.

Instead, then, of creating new dental colleges, let the existing medical schools create new chairs for oral surgery and operative and mechanical dentistry, wherever there is a demand for it. The dentist of the future must have the same primary medical advantages as the physician, afterwards devoting his time and energy more especially to the dental branches. Training in the dental infirmary alone will not fit the dental students for surgical operations on the head or the face, or in the oral cavity. Only a thorough course in the hospital can give him a clear judgment for a right diagnosis.

In recognition of this fact the University of Michigan, the University of Pennsylvania, the Vanderbilt University of Tennessee, and even the University of Maryland have recently established chairs for dental and oral branches.

We are living in a scientific age, and we must prove that we are entitled to the claim of being members of a scientific profession. Let each indivdual answer for himself, and practice his profession on a scientific basis, from a scientific standpoint.

On the other hand, the physician should always bear in mind the effect of his medicines and his treatment on the oral cavity and its contents, and especially the relationship between that and the uterus; how otherwise explain the excruciating pains in apparently sound teeth, occurring during gestation, whether due to the absorption of lime-salts by the fœtus, or to a reflex action between the uterus and the oral cavity? Are not the vaso-motor fibres of the great sympathetic system of nerves widely distributed to the blood vessels? Does not a protracted hyperæmia of the blood vessels of the pulp degenerate the tooth-structure, causing general suffering in the same? Are not diseased teeth conducive to general ill-health—tubercular diseases caused and increased by the foul air eliminated from filthy and diseased parts of the oral cavity? Are not the parasites and monomades in the tartar and fluids of the mouth liable to penetrate the delicate organs of respiration, causing serious difficulties? How many patients have been treated for ozœna and nasal catarrh, when alveolar abscess is the real disease? How often diseased teeth cause serious trouble to the eye and ear?

The physician should therefore have a general knowledge of the theory and practice of dental pathology and physiology and oral surgery; he should be able to recognize when his patients require special dental treatment, and should at least consult with us in regard to it.

Finally, instead of practicing dentistry as a trade, let each one strive for special individual improvement and higher education, reading the journals, participating in the labor of associatons, striving to abolish the prevailing quackery. Then only will we be able to exact recognition from our medical brethren, making dentistry a fruitful branch of the great tree of medicine.

Dr. Salomon further illustrated his position by bringing before the Society a gentleman who had fractured his jaw, two years ago, by lifting a chair with his teeth.

He came for examination four months ago. Removal of all necrosed bone, and an interdental splint and outside plate was advised. This was not accepted, but two months ago the patient placed himself in the hands of two of the most eminent physicians of New Orleans, who, in that time, have merely removed the loose pieces of bone. The fracture is not reduced, and pus is still oozing ireely. This was considered an average specimen of medical practice in the dental field.

DISCUSSION.

*Dr. A. G. Friedrichs* thought the essayist had defined the ideal dentist; that there was much to be learned on both sides; that the physician was less open to censure, proper instruction not being provided by the medical colleges. Only the dentist, who is a medical graduate, can appreciate how little the medical man is taught of dentistry; the time is coming when all medical colleges will have dental departments, going hand in hand, thus attaining our grand ideal. Then we will not have patients who have been treated for facial neuralgia, tic-douloureux, earache, eyeache, *ad infinitum*, until their stomachs were ruined with opium, gelseminum, etc., until, at last, the face swelling, they come to the dentist, and all the trouble is found to come from an abscessed molar, which the dental surgeon correctly diagnoses at the first glance, after the physician has vainly treated for weeks, perhaps months.

*Dr. J. G. McCulloch* thought it proved by the cases cited that medical men had far greater need of a dental education than dentists had of a medical course, the trouble in all of these cases being due to an incorrect diagnosis by the physician, though many dentists meekly yield to the supposed superior wisdom of the physician, and go to him for diagnosis.

*Dr. D. G. Parker* thought that dentists could accomplish much by calling the attention of physicians, among their acquaintance, to such cases, especially in small towns where their influence is greater.

*Dr. Jos. Bauer* thought the dental colleges had been unjustly arraigned for not sufficiently instructing their students; that they had culled from the medical curriculum everything essential to dental science; anatomy, physiology, histology, etc., were thoroughly taught to dental students. So true was this that medical colleges now accepted one term in a dental college as an equivalent in a medical course, while Harvard University would give the degree of M.D. to a graduate of any reputable dental college; that it was not necessary for the dentist to know everything in medicine; that it was an injustice to the noble work done by our dental colleges; that the graduate justly entitled to the degree of D.D.S. should rank as high in the one profession as the M.D. in the other.

*Dr. G. J. Friedrichs* (from the chair) said that the paper opened a wide field in the ethics of both professions; that, as in house building, everything depended on laying a firm foundation; that a man entering the dental profession to-day required a very different preparation from the man who began practice twenty years ago; that a thorough medical course was now the only proper foundation or starting point of a dental education; a man with the degree of M.D. had still all his knowledge of dentistry to acquire, but he would find the medical course invaluable. Physicians were now learning to send their obstinate cases to the dentist. He cited the case of a young lady suffering from gastralgia, headache, and other symptoms; she had become so emaciated that her friends thought she was going into a decline. She was restored to health, increasing in weight from seventy pounds to a hundred and forty-five pounds in three months, as the result simply of having bad teeth extracted, or put in good condition, rendering mastication possible, and a simple course of tonics. He said that statistics showed that, of all the graduates from our dental colleges, not more than five per cent. were found in the profession at the end of ten years; they had gone into something else more rapidly remunerative.

*Dr. O. Salomon* said that the time predicted by Dr. A. G. Friedrichs was nearer than he supposed, as many of the Universities now had dental chairs. He did not consider it necessary for a dental student to take the degree of M.D. also. He was proud to say that after having studied medicine in Germany, he found, on entering the Baltimore Dental College, he did not know anything. The medical colleges which have dental chairs require the regular medical course in anatomy, physiology, etc., with special additional lectures for dental students. Subject passed.

*Dr. Jas. S. Knapp* read a paper entitled:

### CYLINDER FILLINGS.

He made the claim that, of all the forms of gold used for arresting caries, no one was more to be commended than cylinders; that it accomplished, by the wedging process, the most thorough exclusion of air and moisture, by the most expeditious method; that the welding process, in which the cohesive qualities of gold are utilized, is very slow, difficult of adaptation to the borders of the cavity, and involves an amount of pressure dangerous to frail margins and walls, also necessitating the use of the rubber-dam, which not more than ten men in the United States were capable of applying quickly and accurately in cases of more than ordinary difficulty, and which occasions great discomfort, if not absolute pain to the patient, and much worry and discomfiture to the operator. He claimed also that in the use of cohesive gold there is a tendency for the mass to leave the margins and "ball up" towards the centre, occasioning a large percentage of imperfect margins. The cylinders on sale at the dental depots are never suitable for the entire filling; they are only good as "blocks," in connection with other cylinders, of different lengths, sizes, and shapes, and different degrees of hardness, which every successful cylinder operator must be able to make for himself, varying them to suit each case, guided by circumstances and conditions, and the structure of the tooth to be operated on. Some of the cylinders should be cone-shaped, with the larger ends of the first ones at the bottom of the cavity, spreading into the undercuts; alternating the larger ends up and down will cause the cylinders to "dove-tail;" as the cavity fills up a tapering but round instrument must be introduced, piercing between the cylinders, forming space for the introduction of others, the last ones being but little more than a gold wire; hardening the mass by lateral pressure, great density is acquired, and a perfect adaptation to frail margins that would not withstand impaction by any other method. It is true that building out, or contouring, can only be done to very limited extent by this method, but more teeth will be saved than by any other mode of filling. Fancy operators can make greater display of gold, by other methods, exhibiting a living showcase of glistening jewelry, but the duration of contour operations is very brief compared with fillings made with cylinders; the tooth-material contiguous to a contour-filling soon disintegrates from the imperfect margins left. There is no scaling off from the surface of a cylinder filling, each piece extending endways nearly or quite to the bottom of the cavity. A filling which would be worth from $12.00 to $15.00 if made with cohesive gold (from the time necessary to build it out) could be made for, from $5.00 to $7.00 with cylinders, with equal profit to the operator, more comfort to the patient, and greater benefit to the tooth. He did not, however, wish to be understood as conveying more than the idea that for all ordinary cases of filling with gold, cylinders were worthy of the first and highest consideration, though special cases, peculiar conditions, exceptional aptitude or experience would necessarily modify conclusions.

DISCUSSION.

*Dr. O. Salomon* considered that the use of the rubber dam was a great advantage, even in cylinder fillings, and that the use of the "depressed dam" rendered it more easy of application, especially if soap was used instead of waxed floss.

*Dr. A. G. Friedrichs* thought the great advantage of cylinder fillings is in the rapidity with which the work could be done; that in ordinary cavities the gold could be easily impacted in five minutes; that it was not necessary to keep the cavity absolutely dry for a cylinder filling; that if the gold was thoroughly impacted against the walls and bottom of the cavity all moisture would be driven out, as two things could not occupy the same place at the same time; that no matter how dry it might be kept from the exterior, natural fluids oozing from the tubuli would create some moisture. The cylinders, if properly compressed, would perfectly preserve the tooth.

*Dr. O. Salomon* inquired what was supposed to become of the bacteria and other animalculæ of the saliva, if moisture was not excluded. If sealed up they would die, and when dead they must putrify, inevitably creating disturbance.

*Dr. J. R. Knapp* considered many points in the paper extremely extravagant. We must look into underlying principles, and study the natural position of the teeth in the jaw touching each other only at one point near the grinding surface or cutting edge. If gold has to replace tooth-substance it must restore the form nature gave the tooth, a well-known principle recognized by the leading operators of to-day. That although extreme density was not always essential, yet with cylinders it could never be obtained; that portions of a cylinder-filling were liable to loosen and drop from the mass. Another great objection to cylinder filling was the necessity of cutting away tooth-material to make space for placing the cylinders in position and finishing up the filling. True, it is sometimes necessary to cut away some for other methods, but with cohesive gold it could always be restored to the natural form of the tooth. The writer of the paper considered the rubber dam objectionable but the majority of patients prefer it to napkins and wads of absorbent paper saturated with saliva in the mouth. He thought the fillings that could be inserted in five minutes must be very small and very simple and very poorly finished. The essayist cast the imputation on those who gave a perfect finish to their fillings, that it was to make a display of jewelry; but that was not the object—it was to finish the margin finely, to give a hard, dense surface to wear and to keep particles of food from lodging, and generating acids to produce new decay. That one reason why work with cohesive gold was slower was that it was thorough and artistic. With the electro-magnetic mallet gold could be impacted against frail walls where cylinders could not be used without cutting away the enamel; gold under the impact of the electric mallet was extremely malliable, that teeth which had been separated and sawed between would come together again; the teeth would move in their sockets and the whole physiogomy be changed for a lifetime by the malpractice of dentists in using a cross-cut saw between the teeth; the practice could not be too strongly reprobated, causing more injury than any other one thing. We should leave the teeth as God Almighty designed them to be.

*Dr. Jos. Bauer* used cohesive gold, with the electric mallet—could not do without it; thought in many cases it might be well to begin with cylinders and finish with cohesive gold for contouring; did not consider it necessary to restore the contour of approximal surfaces of incisors and canines; when they touched only at the labial or buccal margin with a "V shaped opening" they were self-cleansing and not disfigured, the opening being from the approximo-palatine surface; thought there was much good in the cylinder method and that it would be more used if better known; that a man would be limited in resources who would confine himself entirely to any one method.

*Dr. Geo. J. Friedrichs* (from the chair) said that one reason why cohesive gold is more used is that cylinder filling is not taught in our colleges. Gold can not be so condensed as to resist mastication; even eighteen caret gold will wear down in six months. He cited the case of a patient where eleven teeth were badly worn down; he built them down with cohesive foil, giving a masticating surface of gold. They wore down to such an extent in six months that he rebuilt them, but with the same result. He next made solid gold caps, but with no better result. He then built down the superior cuspid one-fourth inch to open the bite and inserted artificial teeth in regular line with the cuspids. This was done three years ago and the tooth is still good.

There is a force of from three to four hundred pounds in the jaws—fifty pounds will break a filling out of a tooth. Fillings are only temporary at best; the majority will not last ten years; the best, with a good constitution and good care, will not last more than twenty-five years. He was opposed to restoration of contour—or knuckling where occlusion would drive it out of place.

*Dr. J. R. Knapp*, said with reference to the ability of gold to resist mastication that, if properly filled, the gold and the tooth material would wear down evenly with the margins intact.

*Dr. D. G. Parker* said that space could always be obtained by pressure with rubber wedges. He half filled the cavity with cylinders and finished with cohesive gold with the rubber dam; in a frail bicuspid would cut out the entire fissure between the cusps building through with cohesive gold; this would strengthen it so that it would resist any amount of pressure. Subject passed. Dr. W. J. Reese, (Galveston, Texas,) honorary member of the society, read a paper entitled

### URICÆMIA AND ITS EFFECTS UPON THE TEETH.

The following is a brief synopsis :

He was convinced that uric acid in the blood and saliva was the cause of grave trouble in the mouth. Uricæmia is that condition of the system which follows the retention of the excrementitious urinary substances in the blood.

| | | | | |
|---|---|---|---|---|
| Uric acid | $C_{10}$ | $H_4$ | $N_4$ | $O_6$ ; |
| Urea | $6_2$ | $H_4$ | $N_2$ | $O_2$ ; |

The same elements in different proportions. Both are formed from albuminous matters taken in the food in excess of what is required for the nutrition of the system. Muller says uric acid is formed from the white globules of the blood--urea from the red.

Uric acid is found in the excrement of many reptiles, insects and birds Two-thirds of the excrement of the boa-constrictor consists of uric acid. It is always found in the excrement of carnivorous animals, and of the herbivora while sucking, and nourished by a diet rich in nitrogen. Dr. Beale has estimated the quantity of uric acid in healthy human urine to be from one-half to one grain in 1,000 grains ; from five to eight grains being excreted by a healthy adult in twenty-four hours, or .059 grains for every pound of weight of the body. Its chemical character is well marked. To detect its presence, add a drop of nitric acid to the deposit supposed to contain uric acid ; if present, brisk effervescence will take place. Evaporating over a lamp a reddish residue will be left. The addition of a drop of ammonia will give a rich purple tint from the mur-oxide or purpurate of ammonia. Caffeine gives a similar re-action, but is distinguishable by microscopic characteristics. Suspected fluid is to be treated with a few drops of glacial acetic acid, in a watch glass. Immerse in it filaments of silk or tow, and keep it under a glass shade in a warm place from twenty-four to forty-eight hours, when uric acid crystals will be deposited on the filaments.

The presence of uric acid in the saliva was demonstrated in 1881, by Dr. Boucheron, of the Academy of Medicine, Paris.

Saliva is collected on an evaporating dish or watch glass and evaporated over a spirit lamp, adding nitric acid and continuing the evaporation, stirring it from the bottom with a stick dipped in ammonia. Uric acid will be thrown down as a red precipitate.

Uric acid is retained in the blood by diseased action of the kidneys or failure to perform their functions. The presence of uric acid in the blood has been demonstrated by Dr. Garrod to be constant in gout and rheumatism; the kidneys lose their power of excreting uric acid, but eliminate it as in health; less is present in the urine, but during convalescence large quantities are carried off in the urine, and by the emunctories "fever blisters" are also caused through the elimination of uric acid from the blood after fevers.

Alcohol plays an important part in the formation of uric acid in the blood. According to Dr. Beale, large quantities of spirits may exist in the blood for short periods of time, without producing any serious change in the kidneys, but when the renal cells have long been subjected to the influence of blood modified by the constant presence of alcohol they lose their healthy appearance, the liver becoming granular and disintegrated, the kidneys hard, small and wasted. He says the probabilities are that if a hundred persons in good health were subjected to conditions favorable to the production of these results, cirrhosis would result in at least eighty per cent. This condition is sometimes found in very young persons or in persons whose habits of life have been perfectly temperate, even the fœtal kidney being sometimes so affected. These congenital defects are probably nearly always inherited, and lie dormant until developed in febrile and other diseases favorable to the production of the uric acid diathesis. It is thought by many eminent dentists that Pyorrhea Alveolaris is caused by mercurial salivation. This unquestionably aggravates the trouble, but mericurial salivation can be prevented by the administration of an antacid with the mercury, and produced by giving an acid with it. In the same way the presence of an acid existing in the system may cause salivation on the administration of mercury, and this acid may be uric acid. However, less mercury is given now than formerly, while there is more Pyorrhea Alveolaris, the latter occuring when no mercury has been used by the patient or by his ancestors.

Dr. Sansom has estimated the percentage of uric acid in the morning urine, in some diseases, as follows :

| | |
|---|---|
| In acute gout, | .830 |
| Rheumatism, | .802 |
| Heart disease, | .711 |
| Erysipelas, | .679 |
| Excessive debility, | .078 |
| In health | .250 |

It is sometimes so excessive that a drop of nitric acid added to the urine will give abundant amorphous precipitation resembling albumen when first formed, the particles increasing in size till they take the crystalline form of uric acid.

Nasal catarrh, in connection with Pyorrhea Alveolaris is probably produced by the same cause. The hemorrhagic diathesis is also intimately associated with uric acid troubles.*

The effect of uric acid in the blood upon the teeth is a *phagedœna pericementi*—an eating away, or absorption of the peridental membrane. Pyorrhea Alveolaris is a misnomer. Uric acid produces violent inflammation and intense pain; rarely suppurates except in contact with the fluids of the mouth, and not always then, as at the roots of the teeth when protected from the air and the saliva not in contact.

There is sometimes a bony deposit instead of absorption--exostosis. On the palatine root of the superior molar, the gum and alveolus will often be absorbed without suppuration, the labial root being apparently healthy, but exostosed especially if the tooth has no antagonist.

The formation of tophus on the roots of the teeth is a con-

*To check profuse hemorrhage after the extraction of teeth, take a very soft wax impression of the parts, trim it out and fill with a soft batter of plaster and apply immediately, pressing down firmly until the plaster is set. This will succeed when all other means have failed.

comitant of uric acid trouble, but not necessarily so. Absorption of the peridental membrane may take place without deposits, the absorption always preceding the deposit by from one-sixteenth to one-eighth inch.

There is a salivary calculus composed principally of phosphate of lime, sometimes this will be found and also the tophus of uric acid on the same tooth—the phosphate deposit is of a lighter color and more porous. Women, when irregular in menstruation, or during pregnancy and nursing, have in addition to the deposits, the gum festoons purple and bleeding at the slightest touch, the latter feature often the only inconvenience experienced. If not checked pockets will form as in other stages of uricæmic diseases but suppuration rarely takes place. Sometimes, from the quantity of blood and lack of inflammation it constitutes a form of vicarious menstruation. The action of uric acid in dissolving the peridental membrane is the same as in the articulations and periosteum in other parts of the body. The investigations of Dr. Aiguilhen De Serran proves the periosteum and the peridental membrane anatomically the same as the ligaments.

The sytemic treatment is the same as for gout and rheumatism. When the diet has been too largely meat, vegetables should be used freely, as also lime and lemon juice. Alcoholic, vinous, and and malt drinks prohibited. Exercise in the open air, hot baths, keeping the skin healthy, and quinine in small doses.

For local treatment a surgical operation is rarely necessary. The urates dissolve in nitric or acetic acid. The efficacy of peroxide of hydrogen is due to the nitric and muriatic acid used in the manufacture. Uric acid yields its base to nitric acid. Hence the peroxide of hydrogen dissolves the deposits of recent formation and by adding a small quantity of nitric acid the phosphate deposits are also dissolved; the nitric acid unites with the alkaline base. Uric acid tophus can also be dissolved by potash, soda, or ammonia, the alkalies uniting with the uric acid. It has also a great affinity for chloride of sodium which will dissolve the deposits but it is painful to the gums.

A tooth powder composed of prepared chalk and bicarbonate of soda, equal parts with pulverized orris root or South American soap-tree bark, flavored to taste, used freely by the patient, and packed around the teeth at night, in connection with peroxide of hydrogen rubbed briskly over the deposits by the dentist, will remove all accumulations without the use of instruments.

The peroxide must not be exposed to the light. A small quantity should be poured into a clean vessel and applied by means of cotton wrapped on an excavator and saturated. The powder given above is a specific for sensitive dentine near the gums. If the pockets are deep they should be syringed with peroxide of hydrogen and cauterized. Robinson's remedy is very efficacious, the potash neutralizing the uric acid, but should be used with great caution.

℞ Hydrate chloral,
Spts Cochlearea *aa* ℥ *II.*

applied with a mop of cotton on an excavator, or with a camel's hair pencil, will be found equally efficacious and safer for general use. The lips should be protected with a napkin. Passed without discussion.

A paper from Dr. P. J. Friederichs entitled

OBTURATORS

was next read. The writer objected to the usual hard, inflexible obturator made to pass through the opening, with a flange to prevent its slipping from place, as interfering with any efforts of nature towards closing the gap. He thought an artificial velum, of soft pliable elastic material, adapting itself to the varying surface of the soft palate, would frequently allow the aperture to close itself by granulation when thus protected. He described the case of a lady who was obliged to use napkins at the table to remove fluids and food forward through the opening and through the nostrils. The opening was one-quarter of an inch in front of the uvula, and the size of a five-cent piece. She was wearing a partial upper plate. To this, as a temporary appliance, he affixed a springy, strip of eighteen carat gold to which he rivited a disk of rubber dam, which covered the aperture and afforded perfect relief. In a week, however, it became softened and lost its elasticity. Finding it necessary to renew the disk frequently, he devised a method by which the patient could replace the disk at will. Cutting slots in the spring, leaving two dove-tailed flanges and a hole in the disk, it is readily slipped on and held in place by the flanges. Where a plate is not worn an appliance to retain the disk can be attached by clasps to the teeth.

The only points brought out in the discussion of the paper were in corroboration of the statement that openings in both the hard and the soft palate would often close through the unaided efforts of nature if properly protected.

*Dr. J. W. Adams* read a paper entitled

THE ORAL CAVITY AND ITS RELATIONS TO THE GENERAL SYSTEM.

He described in detail the mouth and its several parts—the lips, the tongue, the teeth, the glands, the palate, etc., and their various functions, as speech, taste, mastication, digestion, etc. He traced the intimate connection between the mouth and the nervous, circulatory, and digestive systems, showing that through this connection diseases of the mouth must necessarily affect all other portions of the system, tracing the cause of obscure and persistent diseases, often of portions of the human system the most remote from the mouth, to diseases of the latter; the tongue affording an index to the condition of the stomach; the teeth to the condition of the uterus. Diseases of the blood and of the respiratory organs are caused by the vitiated secretions from carious teeth, chronic abscesses, necrosed bone. He also cited cases of epilepsy, facial neuralgia, amarocis, diseases of the ear and eye, all resulting from affections of the mouth, especially the teeth.

The discussion of this paper only elicited corroborative evidence as cases of gout, hemorrhoids, etc., induced by diseased condition of the mouth and relieved by proper attention to the teeth.

A paper from Dr. A. F. McLain, Santa Rosa, California, was read, entitled

NEW THERAPEUTIC AGENTS.

The remedies enumerated were encalyptus oil, listerine, boracic acid, baro-glyceride, resorcine, and sanitas, all of which have been fully discussed in the journals.

*Dr. J. C. McCulloch* read a paper entitled

AMALGAM.

He spoke of the bitter war waged during its infancy against the advocates of amalgam, so bitter indeed that many operators felt themselves forced to abandon its use in order to maintain their standing in the profession. Many of its once bitterest opponents, however, men who now stand among the representative men of the profession, are now its champions. He said, in substance, that while in the hands of the charlatan and the quack it may be used to deceive and impose upon a confiding public from the ease with which it is manipulated with apparent success by men who have not the skill required to produce good results with gold, nevertheless even the best operators find cavities so located that nothing else can be employed, teeth of a character that no other material will save; and patients whose means will not permit expensive operations in gold. He referred to a recent article published in the *Southern Dental Journal* from the pen of Dr. G. Chisholm, of Columbus, Miss., in which cases of rheumatism, salivation, etc., are attributed to the mercury in amalgam fillings, though not a fact or even a theory is offered to show how or why the amalgam fillings are responsible for the trouble. The essayist humorously suggests that possibly the warm weather caused the mercury to be vaporized, and thus be taken into the system. He also points out the inconsistency of the writer of the article referred to in that, while not doubting that the amalgam fillings were really the cause of all the suffering, the rheumatic pains, salivations, cysts, etc., and otherwise detrimental to health, he nevertheless "fully realizes the merits of amalgam as a filling material."

Amalgam, while allowed no favor, has forced its own way, proved its own virtues, and gained ground steadily, until it now stands second to none when used with judgment and in its proper place. He referred to the statement made in the paper before mentioned—that "amalgam was not expected to preserve teeth a great while," claiming the contrary to be the fact, as proved by the records of our older operators who, years ago, filled teeth with amalgam, which are doing good service to-day. If the amalgam they used preserved the teeth, surely the improved amalgams of to-day will do even more. He also spoke of its great value in the recent methods of use for attaching facial crowns to the remaining roots of natural teeth, by which means hundreds of otherwise useless roots are made to serve a good purpose.

As a means of saving the teeth of the poorer classes, its unrivaled value must be admitted, and this class of sufferers certainly require our sympathy and assistance quite as much as our more wealthy patients. Without amalgam we should see them suffer and hear them cry for aid in vain. If only for them, amalgam is the greatest boon given to dentistry.

DISCUSSION.

*Dr. J. S. Knapp* thought amalgam valuable in cases where the use of gold was impossible, but did not consider it a durable material, but only serviceable for a few years. The chief objection to the use of amalgam lay in the risk of using too much mercury, leaving the amalgam too soft leading to injurious effects. Amalgam fillings being usually "cheap work" the fillings were not properly finished at the cervical margins, nor properly polished when hardened, thus retaining particles of food and creating irritation; also that amalgam had a tendency to render the tooth-substance brittle, causing it to crumble away at the margins.

*Dr. O. Salomon* thought the brittleness observed due to the use of amalgams containing copper. To counteract the contraction of very large amalgam fillings he used pellets of old amalgam left over from the day before, impacted in the new fillings while soft.

*Dr. J. R. Knapp* thought amalgam should be used sparingly on account of its uncertainty—the difficulty of obtaining a reliable article that would produce the same results twice in succession. Sometimes it would bulge, sometimes it would shrink. He would use amalgam very soft in the bottom of large cavities and as dry as possible towards the surface. Amalgam was valuable for partly filling large cavities in the bicuspids or molars, where the cavity extended under the gum, sometimes almost to the alveolus, and where it was next to impossible to so adjust the rubber dam as to permit the use of gold; in such cases the visible portions could be finished with gold, as advocated by N. W. Kingsley. In this case the amalgam should be allowed to harden and then remove with burrs as far as necessary; the matrix would be found of great assistance in all such cases. In amalgam fillings, as in gold, dressing the margins and polishing the fillings are the most essential parts of the operation—not for display, but for service and wear, for the preservation of the tooth and the comfort of the patient. In approximal cavities, where the teeth are close together, gold cannot be finished properly unless tooth-substance is sacrificed. When filled as indicated above, using matrices, little dressing or polishing is required; the material is confined to where it is wanted and there is no surplus to trim off.

*Dr. A. C. Gagle* (New Iberia) thought the combination of gold and amalgam liable to create an electric current, a possible cause of failure.

*Dr. Salomon* said that it had been demonstrated that where the two materials were combined in the same tooth, no current was created.

*Dr. D. G. Parker* (Opelousas) thought gold or even tin cylinders preferable to amalgam; there would be no delay as in waiting for the amalgam to harden; with tin cylinders the rubber dam would not be required; that amalgam would bulge or move and more or less would be left between the teeth below the margins. If gold was added before the amalgam hardened, it would be discolored by the mercury.

*Dr. J. R. Knapp* considered tin an excellent article, but requiring fully as much if not more skill, labor and space to properly handle than gold, in the inaccessible cavities of which he spoke.

*Dr. A. G. Friedrichs* would only use amalgam where absolutely impossible to use anything else, and knew of no such impossibility, except from the inability of the patient to pay for gold. He had experienced all the disadvantages enumerated—the filling leaving the edges of the cavity; the tooth becoming brittle and chipping off at the margins, etc. He preferred to use gold exclusively, and as a rule in the form of cylinders. Does not use the rubber dam. Subject passed.

The President, Dr. G. J. Friedrichs, calling the Vice-President, Dr. E. Telle, to the chair, read a paper entitled

AQUA CALCIS.

He stated that his attention had been called to this subject by reading an article published in the *Independent Practitioner*, November, '85, entitled "Dental Nutrition," in which it was stated that "the administration of Aqua Calcis was efficient in hardening softened decalcified teeth;" and also that "the general diet of the residents of New Orleans, with the cistern water used exclusively for notable purposes, constituting an environment differing considerably from that of any other portion of the country, results in a greater amount and degree of softened decalcified teeth than are to be found elsewhere." That he was led to investigate how far these conclusions accorded with the evidence of others in the same field of labor, as if there was any truth in the assertions made, they ought to be sustained by the observations of other investigators. He found that the general opinion of the best minds in the profession reached the conclusion that "neither diet nor geographical location has anything to do with the formation of the teeth."

In support of this general position he quoted from Magitot that "the nature of drinks used by the population plays but a secondary role, or is perhaps nil;" also from Prof. Truman, to the effect that the old argument from feeding lime to hens to produce shells did not apply in favor of assimilation, as the shells could not be held as organized products, bearing no more relation to the hard tissues of the animal than a calcareous deposit in the mouth to a tooth; that though increased density could undoubtedly be effected by the presentation of food containing the necessary elements, it could not be accomplished by direct administration of the inorganic; that it was a waste of energy to attempt it.

He further offered, in support of the general position, quotations from the paper on the Earthy Phosphates, read by Dr. W. C. Barrett before the American Dental Association at Minneapolis, and from the remarks of Prof. W. H. Morgan made in the discussion of this subject before the Southern Dental Association in New Orleans, as published in the reports of those meetings. Returning to the paper criticised, he did not consider that lack of lime salts caused the poor teeth of residents of New Orleans, as external deposits of phosphates in the form of tartar to such an extent as to require removal to prevent absorption and recession of the gum were not uncommon. He further described the appearance, characteristics, and therapeutic qualities, of lime water and cited Gintshell, Blane, and Alton as to the administration of lime water to dissolve calcareous accretions in the bladder. He said that we have no control over the process of assimilation, the mere presence of the materials being of no value unless appropriated. He considered lime water a most efficacious remedy in neutralizing acidity of the fluids of the oral cavity and the best astringent in flaccidity and sponginess of the gums, preventing putrefaction, and by mild stimulation inducing a healthy reaction of the general tissues of the mouth, in this manner removing the external causes of caries, thus preventing the progress of decay of the teeth. Passed without discussion.

The election of officers resulted as follows:

*Dr. G. J. Friedrichs*, New Orleans, President (re-elected).

*Dr. D. J. Parker*, Opelousas, First Vice-President.

*Dr. M. J. Massingill*, Monroe, Second Vice-President.

*Dr. Chas. Eckhardt*, New Orleans, Recording Secretary (re-elected).

*Dr. A. C. Gagle*, New Iberia, Corresponding Secretary.

*Dr. C. C. Baquie*, New Orleans, Treasurer (re-elected).

EXECUTIVE COMMITTEE.

*Dr. Jos. Bauer*, New Orleans.
" *O. Salomon*, "
" *A. G. Friedrichs*, "
" *J. R. Knapp*, "
" *P. J. Friederichs*, "
" *J. W. Adams*, "

After the usual ceremonies of installation of officers, resolutions of thanks, etc., the Society adjourned to meet in New Orleans on the Wednesday, after Mardi Gras, 1887.

